# Role of proline and GABA in sexual reproduction of angiosperms

**DOI:** 10.3389/fpls.2015.00680

**Published:** 2015-09-04

**Authors:** Marco Biancucci, Roberto Mattioli, Giuseppe Forlani, Dietmar Funck, Paolo Costantino, Maurizio Trovato

**Affiliations:** ^1^Department of Biology and Biotechnology, Sapienza University of RomeRoma, Italy; ^2^Department of Life Science and Biotechnology, University of FerraraFerrara, Italy; ^3^Department of Biology, University of KonstanzKonstanz, Germany

**Keywords:** proline, GABA, pollen, anthers, *P5CS1*, *P5CS2*, Arabidopsis, sexual plant reproduction

## Abstract

Two glutamate derivatives, proline and γ-aminobutyric acid (GABA), appear to play pivotal roles in different aspects of sexual reproduction in angiosperms, although their precise function in plant reproduction and the molecular basis of their action are not yet fully understood. Proline and GABA have long been regarded as pivotal amino acids in pollen vitality and fertility. Proline may constitute up to 70% of the free amino acid pool in pollen grains and it has been recently shown that Arabidopsis mutants affected in the first and rate-limiting step in proline synthesis produce aberrant and infertile pollen grains, indicating that proline synthesis is required for pollen development and fertility. Concerning GABA, a large body of evidence points to this glutamate derivative as a key determinant of post-pollination fertilization. Intriguingly, proline has also been associated with pollination, another aspect of sexual reproduction, since honeybees were reported to show a strong preference for proline-enriched nectars. In this review, we survey current knowledge on the roles of proline and GABA in plant fertility, and discuss future perspectives potentially capable to improve our understanding on the functions of these amino acids in pollen development, pollination, and pollen tube guidance.

## Setting the scene: microsporogenesis and microgametogenesis

**Figure 1:**
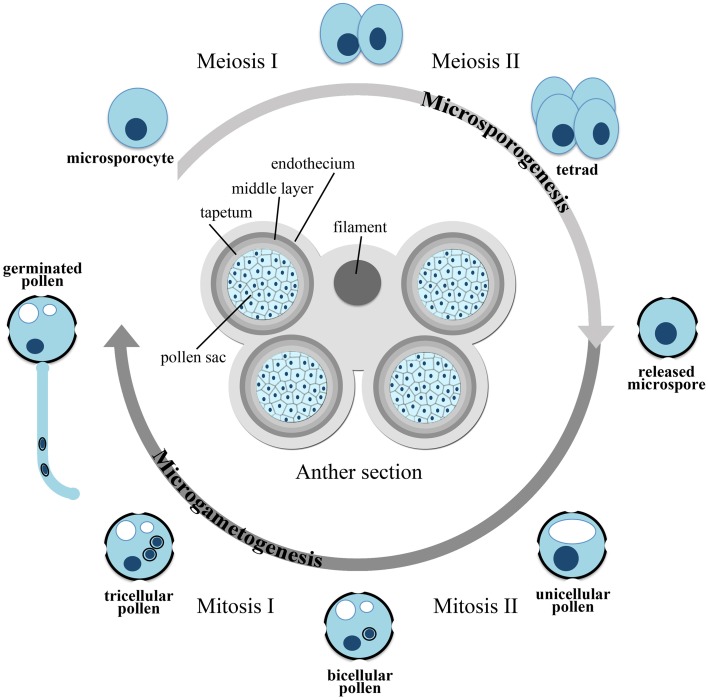
**Scheme of microsporogenesis and microgametogenesis**.

Higher plants cycle between a diploid sporophytic generation and a haploid gametophytic generation. While spending most of their lifespan as diploid individuals, plants rely on their haploid gametophytic generation for sexual reproduction. Cells committed to sexual reproduction undergo meiosis in specialized organs of the flower, called stamens and pistils, and, after a limited number of mitoses, develop as mature haploid gametophytes. The male gametophyte, also referred to as micro-gametophyte or pollen grain, develops in the pollen sac of the anther passing through two sequential developmental phases, called microsporogenesis and microgametogenesis (Figure 1).

In early stages of pollen development, microsporogenesis takes place within the anthers, where archeosporal cells divide periclinally to generate an outer parietal layer and an inner sporogenous layer. The outer layer undergoes both periclinal and anticlinal divisions, to generate concentric layers that differentiate into the endothecium, middle layer, and the tapetum. The inner layer gives rise to the microsporocytes, also known as pollen mother cells or meiocytes, which divide by meiosis to form a tetrad, consisting of four haploid microspores embedded in a joint callose coat. The microspores are eventually released from the tetrad as single-celled haploid microspores, by the action of a ß(1-3) glucanase (callase) secreted by the tapetum. In addition to producing hydrolytic enzymes for callose degradation, the tapetum supplies nutrients and metabolites to the developing microspores and plays an essential role in pollen development, as first shown by Mariani et al. (1990), who reported that a genetic ablation of the tapetal cells results in male sterility. In early stages of pollen development, before meiosis takes place, all the layers of the anther are physically interconnected with the tapetum by plasmodesmata, through which all nutrients and metabolites can be freely exchanged. After tetrad separation, the microspores start to build an extremely durable extracellular layer, the exine, to which the tapetum contributes the major building material, the sporopollenin. However, at the end of microspore development, by the time of pollen mitosis I (PM I), the tapetum starts a process of progressive vacuolization and degeneration, and from this point onward the developing microspores have to rely on endogenous resources. The series of events leading from unicellular microspores to mature bi- or tri-cellular pollen grains, depending on species, is known as microgametogenesis (Figure 1). During this phase the unicellular microspores, generated by callose degradation of the tetrads, begin a process of expansion and vacuolization usually leading to the formation of a large vacuole. The nucleus undergoes PM I, an asymmetric mitotic division, which generates a pollen grain with a larger vegetative cell and a smaller generative cell. The generative cell subsequently separates from the pollen grain wall and gets internalized into the cytoplasm of the vegetative cell. In some plant families (e.g., Cruciferae and Gramineae), the generative cell divides once more by mitosis (PM II) to form two sperm cells in the mature, tri-cellular pollen grain. In many angiosperm families, however, the pollen grain is released from the anther after the first mitotic division as a bi-cellular pollen grain and PM II occurs after pollen germination, when the pollen tube is already growing through the female pistil. The two generative cells are delivered through the pollen tube to the embryo sac within the ovule to fertilize the egg cell and the central cell (double fertilization). At the mature stage, the pollen is covered by the resistant exine coat and is highly dehydrated, probably to withstand even harsh environmental conditions during the transfer to a pistil. Rehydration of the pollen happens on the stigma surface of a compatible pistil and leads to germination and growth of the pollen tube.

## Proline and pollen development: a historical perspective

Beside the well-established role of proline in protein synthesis, this amino acid is known to participate to the set of responses put in place by cells to respond to, and protect from different types of stresses, particularly salt and drought stress (for a review see Hayat et al., 2012). Indeed, accumulation of proline under stress is a well-documented phenomenon in a large number of different organisms, including protozoa, eubacteria, marine invertebrates, algae, and plants (Trovato et al., 2008). In higher plants, however, proline has also been shown to participate in plant development, accumulating under non-stressed conditions in specific developmental phases, particularly in reproductive organs and structures, such as flowers, anthers and pollen grains. The first hints of the special role played by proline in pollen development and function came from qualitative and quantitative analyses of free amino acid content made by independent laboratories in several plant species. As early as 1948, Auclair and Jamieson reported a high, although not precisely quantified, content of free proline in the pollen grains of dandelion (*Taraxum officinale*) and willow (*Salix* spp.) (Auclair and Jamieson, 1948). The first quantitative assessment of proline content in pollen was probably performed by Bathurst (1954), who analyzed the free amino acidic content in the pollen grains of four species of grasses, finding the presence of 17 amino acids, of which proline was the most highly represented accounting for 1.65% of pollen dry weight. Since these initial reports, several other authors (including but not limited to Vansuyt et al., 1979; Venekamp and Koot, 1988; Mutters et al., 1989; Walton et al., 1991; Chiang and Dandekar, 1995; Schwacke et al., 1999) confirmed these findings and reported strong accumulation of proline in floral organs, siliques, and pollen grains of different plant species under unstressed physiological conditions. As evidenced in Arabidopsis by Chiang and Dandekar (1995), the content of proline is low during the vegetative phase, but starts to accumulate dramatically in the reproductive phase, just after floral transition. These authors reported that proline accumulates in *Arabidopsis* reproductive tissues, such as flowers and seeds, to up to 26% of the total free amino acid pool while in vegetative tissues, such as rosette leaves and roots, it only represents 1–3% of the total free amino acids. As pointed out by the authors, proline accumulation in reproductive organs might be correlated to the levels of desiccation tolerance, as most of the tissues enriched in proline, such as pollen grains, seeds and embryos, are naturally dehydrated (Chiang and Dandekar, 1995). In this case proline might function as a compatible osmolyte protecting cells from the detrimental effects of dehydration, and both developmental and environmental responses might be part of a common strategy against drought and water stress. Although there is no general agreement, as yet, on the actual role of proline in development, it is largely accepted that pollen grains are the plant structures with the highest proline concentration. In a study conducted in the anthers of devil's trumpet (*Datura metel*), a *Solanaceous* species, Sangwan (1978) analyzed the changes in the amino acid content occurring in pollen throughout development and found the presence of a limited number of free amino acids (proline, glutamic acid, aspartic acid, threonine, serine, and alanine), of which proline was the most abundant and the only one to show a consistent increment during pollen development. Similarly, during *in vitro* germination of Petunia pollen, Hong-Qi et al. (1982) reported that proline was the most abundant amino acid, accounting for 55% of the total amino acid pool and exceeding by 70% the proline incorporated in proteins (Hong-Qi et al., 1982). In the most striking case, reported by Schwacke et al. (1999), the content of free proline in tomato flowers was found to be 60-fold higher than in any other organ analyzed. Within the floral organs, most of the free proline was found in pollen, where it represented over 70% of total free amino acids.

## Expression pattern of proline metabolic and transporter genes

**Figure 2:**
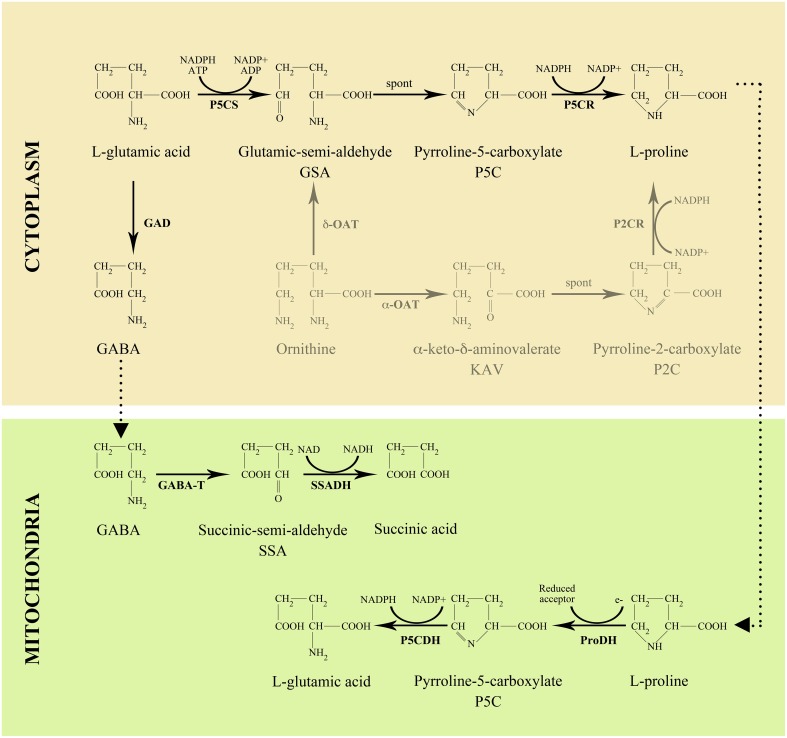
**Pathway of proline and GABA metabolism in higher plants**.

In higher plants, proline is mainly synthesized in the cytoplasm from glutamate (Figure 2), which is converted into glutamic-semialdehyde (GSA) by the bi-functional enzyme δ^1^-pyrroline-5-carboxylate (P5C) synthetase (P5CS). GSA spontaneously cyclizes into P5C, which, in turn, is reduced to proline by the action of P5C reductase (P5CR). Alternative routes of proline synthesis are possible from ornithine *via* ornithine δ-aminotransferase (δ-OAT) and, possibly, ornithine α-aminotransferase (α-OAT), but these alternative pathways have been shown to have no or little relevance in proline synthesis (Funck et al., 2008). The enzyme catalyzing the first and rate-limiting step of proline synthesis in higher plants, P5CS, is coded for in Arabidopsis by *P5CS1* and *P5CS2*, two paralog genes sharing extensive sequence homology (Strizhov et al., 1997). Proline catabolism takes place in mitochondria, where proline is oxidized to glutamate by the sequential action of proline dehydrogenase (ProDH) and pyrroline-5-carboxylate dehydrogenase (P5CDH) (Figure 2). In most plant species several abiotic stresses, particularly salt and drought stress, induce accumulation of high levels of proline. At molecular level stress-induced proline accumulation is mainly due to the increased synthesis and reduced degradation of proline, accounted for by the contemporaneous upregulation of *P5CS1*, and downregulation of *ProDH* (Verbruggen and Hermans, 2008; Szabados and Savouré, 2010). Proline accumulation, however, also occurs under non-stressed conditions in specific developmental phases (Trovato et al., 2008), and is likely controlled by different regulatory conditions. In non-stressed conditions, the pattern of expression of *P5CS1* and *P5CS2* is complex and conflicting data have been reported from different groups. High expression of *P5CS1* was initially detected in Arabidopsis flowers by Savouré et al. (1995) and Yoshiba et al. (1995) by Northern blot analysis. The existence of a second gene coding for P5CS that is subjected to differential regulation, was subsequently assessed by Strizhov et al. (1997), who found high *P5CS1* expression, and, respectively, low *P5CS2* expression in differentiated tissues of Arabidopsis, such as roots, stems, leaves and flowers. Though, the expression of *P5CS2* was high in dividing cells from callus and cell cultures (Strizhov et al., 1997). In contrast, similar levels of both *P5CS1* and *P5CS2* transcripts were reported in flowers and embryos by Mattioli et al. (2009), and in embryos by Székely et al. (2008). However, although *P5CS1* and *P5CS2* transcripts may share a similar pattern of expression in embryos, only homozygous *p5cs2* mutants are embryo-lethal while *p5cs1* mutants are not, indicating that the embryo-lethality of *p5cs2* mutants is not caused by differential transcriptional regulation of the two *P5CS* genes. According to Székely et al. (2008), the striking difference between P5CS1 and P5CS2 in embryo development could be accounted for by a different sub-cellular localization of P5CS1 and P5CS2 proteins in embryonic cells. As observed by these authors, P5CS1-GFP exhibited a peculiar dotted pattern in embryonic cells, in contrast with the uniform cytoplasmic distribution of P5CS2–GFP. By high-resolution confocal laser scanning microscopy, P5CS1-GFP turned out to be sequestered into so far uncharacterized subcellular bodies, suggesting that the sequestered P5CS1 could be a non-functional enzyme unable to complement proline deficiency in homozygous *p5cs2* embryos. Consistent with transcript detection by *in situ*-hybridization, P5CS-GFP was also detected in inflorescence meristems, flower primordia, flower buds and anthers, although experiments with reporter genes should always be interpreted with caution.

Another strong confirmation of the importance of proline in the reproductive phase of angiosperms came from the observation that the genes coding for metabolic enzymes and transport proteins of the proline pathway (Figure 2) tend to be highly expressed in reproductive organs. Rentsch et al. (1996) reported that *Proline Transporter 1* (*ProT1*), a gene coding for a specific proline transporter belonging to the amino acid permease (AAP) gene family, was highly expressed in Arabidopsis flowers, and was downregulated in the course of flower development, suggesting a role of *ProT1* in flowers. Similarly Schwacke et al. (1999) reported that *LeProT1*, a tomato homolog of *ProT1*, was highly expressed in tomato flowers, as revealed by Northern blot analysis. The expression of *LeProT1* increased steadily during flower development, and reached a peak at anther dehiscence. Moreover, through *in situ* hybridization performed on dissected flowers, these authors found that the expression of *LeProT1* is restricted to anthers, consistent with the high concentration of proline measured in pollen grains. More recently Grallath et al. (2005) and Lehmann et al. (2011) reported that *AtPro1*, as well as *AtPro3*, a new member of the *AtProT* gene family, were strongly expressed in flowers, especially in mature pollen, suggesting that AtProT1 is important for flower development. In addition, *AtProT1* expression was detected in the phloem or phloem parenchyma cells throughout the whole plant, indicating a possible role in long-distance proline transport. Interestingly, Grallath et al. (2005) also described that, contrary to previous reports, all ProT amino acid transporters could transport γ-aminobutyric acid (GABA), in addition to proline, although with lower efficiency. This finding raises the possibility that GABA as well as proline might be transported to floral tissues through the phloem.

Surprisingly, deletion of the gene *ProT1* in Arabidopsis had no effect on the composition of the free amino acid pool in mature pollen, while it affected the contents of free glutamate and arginine, but not proline or GABA in germinating pollen (Lehmann et al., 2011). In addition, in spite of the altered concentrations of glutamate and arginine, pollen germination of *prot1-1* mutants was normal, possibly because of a functional complementation by *ProT3.* Unfortunately, amino acid analyses of double or triple *prot* mutant pollen have not been reported yet.

The evidence that the variation of glutamate and arginine was only seen in germinating pollen from *prot1-1* mutants may suggest that proline transport is especially or uniquely active during pollen germination. This hypothesis is consistent with independent pollen transcriptome analyses, which detected high *ProT1* transcript abundance during late stages of pollen maturation, just before pollen germination (Honys and Twell, 2004; Bock et al., 2006). The variation of the amino acid composition of *prot1-1* germinating pollen relative to wild type—with glutamate and arginine showing a lower and higher concentration, respectively—is more difficult to interpret. According to Shelp et al. (1999) *in vitro* germination and subsequent harvesting is stressful to germinating pollen and causes a raise of proline and GABA. Accordingly, we may speculate that under stress conditions, *ProT3* may not be able to compensate for the lack of ProT1 in germinating *prot1-1* pollen, causing a compensative induction of endogenous proline synthesis and, in turn, depletion of glutamate and increase of arginine.

In Arabidopsis, the genes coding for the enzymes mediating proline catabolism are also highly expressed in reproductive organs, and both *ProDH* and *P5CDH* are more expressed in floral tissues, especially pollen, than in vegetative tissues. In 8-week-old Arabidopsis plants expressing the gene *uidA* under the control of the *ProDH* promoter, GUS expression was observed in pollen, in mature stigmas with pollen, in stigma of immature siliques, in ovules, and in the abscission zone of sepals and petals (Nakashima et al., 1998). Similarly, strong GUS expression was detected in pollen grains of P5CDH-GUS transgenic Arabidopsis by Deuschle et al. (2004). In spite of the high level of *ProDH* and *P5CDH* transcription driven in pollen by their respective promoters, the actual activities of these enzymes have not been analyzed to date, and their putative function in pollen remains unknown.

## New insights in the role of proline in pollen development

Recently, Arabidopsis mutants defective in proline biosynthetic enzymes were found to produce aberrant and infertile pollen grains, providing genetic and molecular support to the idea that proline is required for pollen development (Funck et al., 2012; Mattioli et al., 2012). T-DNA insertional mutants have been characterized for both *P5CS1* (Mattioli et al., 2008; Székely et al., 2008) and *P5CS2* (Székely et al., 2008; Mattioli et al., 2009), and their phenotypes provided information about the specific functions of P5CS1 and P5CS2 that have been summarized previously (Szabados and Savouré, 2010).

Homozygous mutants for *p5cs2* are embryo lethal under normal cultivation conditions (Székely et al., 2008; Mattioli et al., 2009), although Funck et al. (2012) were able to generate viable and fertile homozygous *p5cs2* mutants by *in vitro* cultivation of immature mutant seeds supplemented with exogenous proline and sucrose. In contrast, *p5cs1 p5cs2* double mutants, homozygous for both *p5cs1* and *p5cs2* mutant alleles, were not vital and could not be isolated. However, by using heterozygous double mutants, two groups independently reported defects in pollen development and fertility associated with proline deficiency in presumably double homozygous pollen (Funck et al., 2012; Mattioli et al., 2012). Both groups found that in reciprocal crosses between plants homozygous for *p5cs1* and heterozygous for *p5cs2* (*p5cs1 p5cs2/P5CS2*) and wild type, the *p5cs2* mutant allele was not transmitted to the offspring when the proline-deficient *p5cs1 p5cs2/P5CS2* plant was used as a pollen donor, but was detected in almost half of the progeny when *p5cs1 p5cs2/P5CS2* flowers were used as a female. In addition, the dominant sulfadiazine resistance marker, linked to the *p5cs2* allele, was found to segregate in a 1:1 ratio, as expected in a gametophytic mutation, and the siliques of the self-pollinated population were found to be devoid of aberrant embryos, as expected if a gametophytic defect should prevent the formation of homozygous embryos (Funck et al., 2012; Mattioli et al., 2012). Morphological and functional analysis of the mutant pollen revealed, at least from stage 11 of pollen development, a population of small, progressively degenerating pollen grains, representing about half of the total pollen population. The aberrant pollen grains appeared misshaped, shriveled and no longer viable, as revealed by orcein staining, DAPI analysis, Alexander's staining, and *in vitro* pollen germination assays. As expected for a mutant carrying mutations in both the genes encoding P5CS, the partial double mutant *p5cs1 p5cs2/P5CS2* accumulates very little proline, on average one fourth as much as a wild type, and this proline shortage is the likely cause of the developmental defects of pollen grains from *p5cs1 p5cs2/P5CS2* mutants. Accordingly, the content of free proline measured in pollen grains from *p5cs1 p5cs2/P5CS2* mutants was 105 ± 23 ng, roughly corresponding to 15 pg/pollen of proline, compared to 336 ± 31 ng, roughly corresponding to 48 pg/pollen, measured in pollen grains from Col-0 wild type (Mattioli et al., 2012). Importantly, 10 μM L-proline, supplemented *in planta* to developing anthers of *p5cs1 p5cs2/P5CS2* mutants, significantly complemented the morphological and physiological defects of pollen grains from *p5cs1 p5cs2/P5CS2* mutants (Mattioli et al., 2012). Surprisingly, pollen carrying a mutation in the single copy of *P5CR*, the gene coding for the second and final step in proline biosynthesis, did not show developmental or fertility defects (Funck et al., 2012). Although proline is clearly required for pollen development and fertility, the relative role of proline coming from, or synthesized in, gametophytic (i.e., pollen grains) and sporophytic (i.e., tapetum) tissues is yet to be clarified. Intriguingly the presence of the *p5cs2* mutant allele was detected, by PCR analysis, also in the wild type-like population of pollen grains from *p5cs1 p5cs2/P5CS2* mutants (Mattioli et al., 2012), suggesting that the phenotypic aberrations observed in pollen grains from *p5cs1 p5cs2/P5CS2* flowers, may not, or not only, have a genetic basis, which prompts an investigation of the relevance of proline coming from either sporophytic or gametophytic tissues. Equally unknown, at present, is the role of proline in pollen development. Several authors (Chiang and Dandekar, 1995; Székely et al., 2008) have proposed that proline accumulation may function to protect macromolecular structures in pollen grains during dehydratation, essentially because proline is a well-known compatible osmolyte and because a mature pollen grain is a highly dehydrated structure. Although this is a likely hypothesis, it still lacks a formal demonstration, and other functions, such as energy source or signaling molecule, are equally possible and are being actively investigated.

## Proline and pollination: to bee or not to bee

Intriguingly, proline has been also associated with pollination, another aspect of sexual reproduction of angiosperms, since honeybees were reported to show a strong preference for proline-enriched nectars (Carter et al., 2006; Bertazzini et al., 2010). To ensure efficient pollination, numerous angiosperms depend upon pollinators, usually birds or flying insects, to transfer the mature gametophyte from the dehisced anther to a receptive stigma. Pollinators are attracted to pollen grains by the use of specialized strategies co-evolved with prevalent pollinator species. In most of the cases flowers have evolved bright and vivid colors, fragrant scents and sweet and nourishing nectars to attract insects, particularly butterflies and honeybees. Once a pollinator lands on a flower, during its daily foraging activity, pollen grains get stuck on the pollinator to be transported from flower to flower to accomplish a high degree of cross-pollination. However, many of the pollinating insects also actively collect the nutrient- and proline-rich pollen itself, especially to support their own reproduction and growth. By collecting a significant proportion of the pollen, the pollinators force the plants to produce pollen in large excess over the need for pollination.

Key to pollination activity is nectar, an aqueous sugar solution mainly derived from phloem sap and synthesized in specialized cells, located at the base of the ovary and known as nectaries. Within these specialized floral cells, phloem sap is enzymatically processed and transformed into nectar. A relevant fraction of the sucrose present in the phloem, for instance, is usually hydrolyzed to glucose and fructose. However, not all the components of the phloem sap are modified and in several instances nectar composition simply reflects the composition of phloem sap, as in the case of proline, which mainly derives from the proline present in phloem sap and actively transported from vegetative to reproductive tissues.

The molecular and genetic basis of the dynamic interaction between pollinators and proline content in nectar is unknown, but it might be mediated by pollination signals similarly to the coordination between pollination and nectar production, which has been shown to be coordinated either by jasmonic acid (Heil, 2004; Radhika et al., 2010) or auxin (Bender et al., 2013). Regardless of environmental cues, nectar production and secretion are under strict developmental control to ensure coordination between pollen shed and nectar secretion and to minimize energy waste. It has been calculated that nectar production consumes up to 37% of a plant's available energy (Pyke, 1991). Consistently a large number of genes involved in regulating nectar composition and secretion are timely expressed in nectaries or adjacent tissues. As an example, immature nectaries are devoid of invertase and accumulate starch in early developmental phases. Around anthesis *CELL WALL INVERTASE 4* (*AtCWINV4*), becomes strongly induced in nectaries allowing them to accumulate the glucose and fructose necessary for nectar production (Ruhlmann et al., 2010). Accordingly, because proline homeostasis derives from a fine-tuning among proline synthesis, degradation and transport, the production of nectars enriched in proline likely implies a complex developmental and tissue-specific regulation of proline metabolism. Even if proline is predominantly delivered by the phloem sap, a contribution of local proline synthesis in the nectaries from glutamate cannot be role rule out. In support of this possibility, microarray and RNA sequencing data from Arabidopsis nectaries revealed significant expression of *P5CS2* in mature nectaries (Kram et al., 2009; Bender et al., 2013).

Honeybees, the most efficient and wide-spread pollinators on a world scale, use nectar as their primary carbohydrate, amino acid and energy source, to provide energy for flight, colony maintenance, general daily activities and nutrient storage for the winter. There is an intimate relationship between nectar and honeybees as, on one hand, nectar is essential for honeybee's life and, on the other hand, plants need the foraging activity of honeybees for cross-pollination, and eventually species survival. The attractiveness of nectars for honeybees has important practical implications since a more attractive nectar will induce bees to show a higher fidelity to flowers of the same species and more cross-fertilized seeds will be produced, increasing, in turn, crop productivity (Allen-Wardell et al., 1998). The importance of pollinator-driven cross-fertilization might be questioned for the survival of autogamous plant species, such as Arabidopsis. However, despite the advantages of self-pollination over cross-pollination in some ecological niches, occasional cross-pollination is still advantageous even in autogamous species, most of which are facultative autogamous plants (Goodwillie et al., 2005).

In addition, the quality and quantity of the honey, which bees produce from nectar, is critically dependent on the nectars produced by different plant species. Not all nectars, however, are equally attracting and nutritive, and marked differences exist among nectars from different plant species, which deeply orient honeybee's foraging activities to selected plant species. Nectar composition is extremely variable and is mainly composed of sugars, especially sucrose, glucose and fructose (Baker and Baker, 1973; Jackson and Nicolson, 2002), as well as of a number of other compounds including proteins, lipids, organic acids, alkaloids, phenolics, terpens, flavonoids, vitamins, and amino acids (Ball, 2007). Nectars also contain specific plant-defense proteins that are thought to protect the gynoecium from microbial invasion brought about by pollinators and wind (Peumans et al., 1997; Carter et al., 1999; Carter and Thornburg, 2000, 2004; Thornburg et al., 2003; Naqvi et al., 2005). Although the amino acid pool is a minor component of the nectar sap, ranging from 0.002 to 4.8% of organic matter (Gardener and Gillman, 2001), it is of relevant biological importance, at least for insects, and it has been shown that nectars from plants pollinated by insects have higher amino acid concentration than nectars from plants pollinated by birds (Baker and Baker, 1986). In addition, the quality and quantity of nectar's amino acids are believed to enhance insect longevity and fecundity (Mevi-Schutz and Erhardt, 2005). Proline, in particular, has been shown to be the most abundant amino acid in the nectar of many angiosperms (Gardener and Gillman, 2002; Kaczorowski et al., 2005; Carter et al., 2006; Terrab et al., 2007). Furthermore, it has been reported that honeybees exhibit a strong preference toward nectars rich in the amino acid proline. According to Carter et al. (2006), honeybees prefer proline-rich nectars over nectars lacking or poor in proline, and can specifically perceive and track proline-rich nectars or sugar solutions supplied with proline. The honeybees' ability to “taste” proline relies on the stimulation of the salt cells of labellar chemosensory cells, and equivalent cells are found on the sensory apparatus of further insect taxa, including hymenoptera and lepidoptera (Hansen et al., 1998; Gardener and Gillman, 2002). The strong preference that honeybees seem to grant to proline-rich nectars may rely on the energy property of this amino acid, which has been proposed as the main fuel employed by honeybees during the earliest or more expensive stages of flight (Micheu et al., 2000). Indeed, the complete oxidation of one molecule of proline yields 21 ATP molecules, almost as many as the 24 ATP molecules obtained from complete oxidation of one molecule of glucose, but obtained with no initial ATP consumption (Micheu et al., 2000). Bertazzini et al. (2010) have confirmed and extended these reports by analyzing, by dual choice feeding tests, the preference of forager honeybees for specific amino acids. They found that honeybees prefer artificial nectar containing proline over artificial nectar containing only sugars. On the contrary, nectars containing either alanine or serine gave neutral or negative responses. Importantly, when honeybees were given the choice between nectars supplemented with different amino acids, proline was preferred above both alanine and serine, and alanine above serine. While sugars (or generally energy and organic carbon) are usually not a limiting resource for plant growth, the nitrogen contained in amino acids usually is. More detailed studies will be required to reveal whether plants enrich their nectar in proline to make it especially nutritious and thus attractive for insects, or whether they try to cheat on the insects by specifically enriching proline as the amino acid that insects can best detect in the nectar.

## GABA and pollen tube guidance

Another glutamate derivative critically important for plant reproduction is GABA, a non-proteinogenic amino acid ubiquitously present in plants and implicated in pH regulation, plant development and pathogen defense (Shelp et al., 1999). GABA is also regarded as a stress molecule involved in the metabolic responses of plants to either abiotic stresses (Mazzucotelli et al., 2006; Michaeli and Fromm, 2015), such as low light (Michaeli et al., 2011) and salt (Renault et al., 2013), or pathogen attack (Forlani et al., 2014).

GABA is directly produced from glutamate in the cytosol by the action of glutamate decarboxylase (GAD; Figure 2). GABA degradation takes place in the mitochondrion where it is converted to succinic acid by the sequential action of GABA transaminase (GABA-T) and succinic semialdehyde dehydrogenase (SSADH) (Shelp et al., 1999). A large body of evidence points to GABA as a molecule involved, at least in some species, in pollen tube growth and guidance. In flowering plants, after pollen grains are deposited on the stigma surface of a diploid female pistil, the pollen starts germination and begins to elongate a pollen tube, a cytoplasmic protrusion through which the two male sperm cells are polarly transported to the elongating tip and finally delivered to the embryo sack. Unlike animal sperms and spermatocytes of primitive plants, such as bryophytes, pollen grains from higher plants have lost their flagella during evolution, and cannot swim anymore, relying completely on pollen tube elongation and intracellular transport to reach the embryo sac and fulfill fertilization. The elongation of the pollen tube starts at a receptive stigma, continues along the style through several different cell layers, and is eventually guided to an ovule micropyle, to enter the ovule. Once the tip of the pollen tube has reached the target, the two sperm cells are released to fuse with the embryo sac, and eventually produce a diploid zygote and the triploid endosperm nucleus. As inferred by the number of mutants affecting pollen guidance (Higashiyama and Takeuchi, 2015), this developmental process is critical for plant reproduction, and of unexpected complexity. Although a simple mechanical guidance has been reported in some species (Higashiyama and Takeuchi, 2015), pollen tubes are usually guided to their target by a number of signal and attractant molecules, such as Ca^++^ (Iwano et al., 2014), lipids (Wolters-Arts et al., 1998), pollen coat proteins (Mayfield et al., 2001), arabinogalactans (Wu et al., 2000; Suárez et al., 2013), pectins (Mollet et al., 2000), secreted cystein-rich proteins (Okuda et al., 2009), or brassinosteroids (Vogler et al., 2014). Consistently, transcriptomic analyses carried out both in *Arabidopsis thaliana* and in *Torenia fourneri* pollen tubes have discovered an extraordinarily high number of genes involved in pollen tube elongation, and, above all, that pollen tubes modify gene expression profiles during their growth along the pistil (Qin et al., 2009; Okuda et al., 2013).

A novel role for GABA in pollen tube growth and guidance was found out by Palanivelu et al. (2003) by characterizing the *A. thaliana POP2* gene, coding for a GABA-T. Previously, the *pop2* mutation was observed to cause a late-stage defect in the elongation of the pollen tube, which bypassed the micropyle and thus failed to hit the ovule cells (Wilhelmi and Preuss, 1996). Consistently with a role of POP2 in GABA degradation, *pop2* flowers exhibited GABA accumulation—as much as 60 fold higher in *pop2-1* flowers than in wild type flowers—leading to infertility because *pop2* pollen tube growth was arrested or misguided in *pop2* pistils. Fertility rate and floral GABA levels were found inversely correlated across different *pop2* alleles (Palanivelu et al., 2003) suggesting that the infertility of the *pop2* homozygous mutants might be associated with an aberrant accumulation of GABA along the style. Interestingly, however, wild type pollen was able to fertilize flowers of *pop2* mutants, indicating that the egg and other embryo sac cells are functional. The higher success rate of wild type over *pop2* mutant pollen additionally indicates that GABA processing by GABA-T within the pollen tube is essential for guidance toward the embryo sack. By a combination of enzymatic and immunohistochemical methods, a GABA gradient, with increasing concentrations from stigma to micropyle, was identified in wild type pistils, but not in *pop2* pistils that showed steadily high levels of GABA all along the pistil. In addition, in *in vitro* germination assays, a large excess of exogenous GABA was inhibitory to pollen tube growth, although at low concentrations GABA had a stimulatory effect (Palanivelu et al., 2003). For wild type, GABA concentrations ranging from 1 to 10 mM stimulated pollen tube elongation, while higher concentrations were inhibitory. In contrast, *pop2* pollen tubes were stimulated at GABA concentrations as low as 1–10 μM, and were inhibited at concentrations higher than 10 μM (Palanivelu et al., 2003). The establishment of a correct GABA gradient, therefore, is necessary to assist fertilization by guiding the growing pollen tube from the stigma surface to the ovule micropyle, in a way that resembles the guidance function of GABA in animal systems, where the migrating neuronal precursor cells of spinal and cortical neurons reach their target destinations with the help of GABA receptors and Ca^++^ signaling (Behar et al., 2001).

As in animals, the mechanism of GABA guidance of pollen tube seems in fact to rely on Ca^++^ signaling. Yu et al. (2014) measured the dynamics of Ca^++^ fluxes, by means of whole-cell voltage-clamp experiments and non-invasive micromeasurement technology, and proposed that the influx of Ca^++^ increased in pollen tubes in response to exogenous GABA. In addition, since the Ca^++^ responses to GABA were specifically blocked by Gd^3+^, a specific inhibitor of Ca^++^-permeable channels, the authors concluded that signaling triggered by exogenous GABA affects pollen tube growth by modulating putative Ca^++^-permeable membrane channels.

Recently, the activity of an aluminum-activated malate transporter in wheat (TaALMT1, Ramesh et al., 2015), belonging to a large family of plant anion channels/transporters (ALMT) was shown to be regulated by GABA. ALMTs of six plant species were regulated by GABA, confirming that GABA acts as a signaling molecule by regulating ion fluxes across cell membranes in plants, as it does in animals. Ramesh et al. (2015) demonstrated that malate flux through TaALMT1 is stimulated by anions and inhibited by GABA leading to increased or reduced root growth, respectively, most probably mediated by changes in the membrane potential. Interestingly, malate flux and, in turn, root growth were specifically inhibited by muscimol—a GABA analog working as a specific agonist of mammalian GABA_A_ receptors (Olsen and Sieghart, 2008). In addition, the effect of muscimol was attenuated by treatment with bicuculline—a well-known competitive antagonist of GABA binding to mammalian GABA_A_ receptors (Olsen and Sieghart, 2008).

Overall these results indicate that GABA exerts at least some of its multiple physiological effects in plants, including pollen tube guidance and root growth, through modulation of ALMT activity, and that GABA can finally be regarded as a genuine signaling molecule in plant, as it is in animals. Accordingly Ramesh et al. (2015) provided evidence that also pollen tube elongation and guidance depend on GABA-gated activity of ALMTs, since muscimol reduced *A. thaliana* and *Vitis vinifera* pollen tube elongation *in vitro*, while bicuculline antagonized muscimol regulation.

## Conclusions and future perspectives

The overall picture that emerges from this review is the surprising and specific importance of proline and GABA in aspects of plant reproduction as important as pollen development, pollination, and pollen tube guidance. Egg cell development seems much more tolerant to disturbances in proline and GABA metabolism, potentially because the embryo sack is in closer contact to the parental tissue. For each one developmental process, however, much work has still to be done and we have more open questions than precise answers available. For instance, during pollen development and fertilization the importance of proline synthesized within the male gametophyte, from unicellular microspore to tricellular pollen, compared to proline that may be provided by sporophytic tissues, is still unclear. In addition, the precise function of proline in pollen development and fertility remains, as yet, elusive.

With regard to the role of proline in pollination, by contrast, it is generally believed that the high energetic value of proline has been the underlying reason for the actual preference of honeybees for proline-rich nectars. It remains to be seen, however, how strong this preference is, and its ecologic and agronomic impact on crops and cultivations.

As to the role of GABA in pollen guidance, in spite of the recent advances of our knowledge on GABA signaling, further work is still needed in diverse areas of research. A major field of interest will be the identification and characterizations of novel activators, agonists and antagonists of plant ALMTs as well as of second messengers and downstream genes, to hopefully improve our knowledge of GABA signaling and to explore the possibility of manipulating GABA-mediated developmental processes. Equally important would be the crystallization of the ALMT receptor either free or bound to GABA or GABA analogs to understand the action of these drugs and the underlying mechanism of GABA signaling. Finally, the question emerges whether or not the functions of the glutamate-derived amino acids proline and GABA in reproduction may be interconnected. Further work is needed to address these fascinating questions.

## Author contributions

All authors have contributed significantly and have approved the final manuscript.

### Conflict of interest statement

The authors declare that the research was conducted in the absence of any commercial or financial relationships that could be construed as a potential conflict of interest.

## References

[B1] Allen-Wardell G. Bernhardt P. Bitner R. Burquez A. Buchmann S. Cane J. (1998). The potential consequences of pollinator declines on the conservation of biodiversity and stability of food crop yields. Conserv. Biol. 12, 8–17. 10.1046/j.1523-1739.1998.97154.x

[B2] Auclair J. L. Jamieson C. A. (1948). A qualitative analysis of amino acids in pollen collected by bees. Science 108, 357–358. 10.1126/science.108.2805.357 17810996

[B3] Baker H. G. Baker I. (1973). Amino acids in nectar and their evolutionary significance. Nature 241, 543–545. 10.1038/241543b04693956

[B4] Baker H. G. Baker I. (1986). The occurrence and significance of amino acids in floral nectar. Plant Syst. Evol. 151, 175–186. 10.1007/BF02430273

[B5] Ball D. W. (2007). The chemical composition of honey. J. Chem. Educ. 84, 1643–1646. 10.1021/ed084p164326314562

[B6] Bathurst N. O. (1954). The amino acids of grass pollen. J. Exp. Bot. 5, 253–256. 10.1093/jxb/5.2.25326221749

[B7] Behar T. N. Smith S. V. Kennedy R. T. McKenzie J. M. M. Maric I. Barker J. L. (2001). GABA_B_ receptors mediate motility signals for migrating embryonic cortical cells. Cereb. Cortex 11, 744–753. 10.1093/cercor/11.8.744 11459764

[B8] Bender R. L. Fekete M. L. Klinkenberg P. M. Hampton M. Bauer B. Malecha M.. (2013). *PIN6* is required for nectary auxin response and short stamen development. Plant J. 74, 893–904. 10.1111/tpj.12184 23551385

[B9] Bertazzini M. Medrzycki P. Bortolotti L. Maistrello L. Forlani G. (2010). Amino acid content and nectar choice by forager honeybees (*Apis mellifera* L.). Amino Acids 39, 315–318. 10.1007/s00726-010-0474-x 20091414

[B10] Bock K. W. Honys D. Ward J. M. Padmanaban S. Nawrocki E. P. Hirschi K. D.. (2006). Integrating membrane transport with male gametophyte development and function through transcriptomics. Plant Physiol. 140, 1151–1168. 10.1104/pp.105.074708 16607029 PMC1435806

[B11] Carter C. Graham R. A. Thornburg R. W. (1999). Nectarin I is a novel germin-like protein expressed in the nectar of *Nicotiana* sp. Plant Mol. Biol. 41, 207–216. 10.1023/A:1006363508648 10579488

[B12] Carter C. Shafir S. Yehonatan L. Palmer R. G. Thornburg R. (2006). A novel role for proline in plant floral nectars. Naturwiss 93, 72–79. 10.1007/s00114-005-0062-1 16365739

[B13] Carter C. Thornburg R. W. (2000). Tobacco Nectarin I: purification and characterization as a germin-like manganese superoxide dismutase implicated in the defense of floral reproductive tissues. J. Biol. Chem. 275, 36726–36733. 10.1074/jbc.M006461200 10952990

[B14] Carter C. Thornburg R. W. (2004). Is the nectar redox cycle a floral defense against microbial attack? Trends Plant Sci. 9, 320–324. 10.1016/j.tplants.2004.05.008 15231276

[B15] Chiang H. H. Dandekar A. M. (1995). Regulation of proline accumulation in *Arabidopsis* during development and in response to dessication. Plant Cell Environ. 18, 1280–1290. 10.1111/j.1365-3040.1995.tb00187.x

[B16] Deuschle K. Funck D. Forlani G. Stransky H. Biehl A. Leister D.. (2004). The role of δ^1^-pyrroline-5-carboxylate dehydrogenase in proline degradation. Plant Cell 16, 3413–3425. 10.1105/tpc.104.023622 15548746 PMC535882

[B17] Forlani G. Bertazzini M. Giberti S. (2014). Differential accumulation of γ–aminobutyric acid in elicited cells of two rice cultivars showing contrasting sensitivity to the blast pathogen. Plant Biol. 16, 1127–1132. 10.1111/plb.12165 24521266

[B18] Funck D. Stadelhofer B. Koch W. (2008). Ornithine-δ-aminotransferase is essential for arginine catabolism but not for proline biosynthesis. BMC Plant Biol. 8:40. 10.1186/1471-2229-8-40 18419821 PMC2377265

[B19] Funck D. Winter G. Baumgarten L. Forlani G. (2012). Requirement of proline synthesis during Arabidopsis reproductive development. BMC Plant Biol. 12:191. 10.1186/1471-2229-12-191 23062072 PMC3493334

[B20] Gardener M. C. Gillman M. P. (2001). Analyzing variability in nectar amino acids: composition is less important than concentration. J. Chem. Ecol. 27, 2545–2558. 10.1023/A:1013687701120 11789958

[B21] Gardener M. C. Gillman M. P. (2002). The taste of nectar—a neglected area of pollination. Oikos 98, 552–557. 10.1034/j.1600-0706.2002.980322.x

[B22] Goodwillie C. Kalisz S. Eckert C. G. (2005). The evolutionary enigma of mixed mating systems in plants: occurrence, theoretical explanations, and empirical evidence. Annu. Rev. Ecol. Evol. Syst. 36, 47–79. 10.1146/annurev.ecolsys.36.091704.175539

[B23] Grallath S. Weimar T. Meyer A. Gumy C. Suter-Grotemeyer M. Neuhaus J.-M.. (2005). The AtProT family. Compatible solute transporters with similar substrate specificity but differential expression patterns. Plant Physiol. 137, 117–126. 10.1104/pp.104.055079 15618414 PMC548843

[B24] Hansen K. Wacht S. Seebauer H. Schnuch M. (1998). New aspects of chemoreception in flies. Ann. N.Y. Acad. Sci. 855, 143–147. 10.1111/j.1749-6632.1998.tb10556.x 9929595

[B25] Hayat S. Hayat Q. Alyemeni M. N. Wani A. S. Pichtel J. Ahmad A. (2012). Role of proline under changing environments. Plant Signal. Behav. 7, 1456–1466. 10.4161/psb.21949 22951402 PMC3548871

[B26] Heil M. (2004). Induction of two indirect defences benefits Lima bean (*Phaseolus lunatus*, Fabaceae) in nature. J. Ecol. 92, 527–536. 10.1111/j.0022-0477.2004.00890.x

[B27] Higashiyama T. Takeuchi H. (2015). The mechanism and key molecules involved in pollen tube guidance. Annu. Rev. Plant Biol. 66, 393–413. 10.1146/annurev-arplant-043014-115635 25621518

[B28] Hong-Qi Z. Croes A. F. Linskens H. F. (1982). Protein synthesis in germinating pollen of Petunia: role of proline. Planta 154, 199–203. 10.1007/BF00387864 24276061

[B29] Honys D. Twell D. (2004). Transcriptome analysis of haploid male gametophyte development in Arabidopsis. Genome Biol. 5:R85. 10.1186/gb-2004-5-11-r85 15535861 PMC545776

[B30] Iwano M. Igarashi M. Tarutani Y. Kaothien-Nakayama P. Nakayama H. Moriyama H.. (2014). A pollen coat- inducible autoinhibited Ca^2+^-ATPase expressed in stigmatic papilla cells is required for compatible pollination in the Brassicaceae. Plant Cell 26, 636–649. 10.1105/tpc.113.121350 24569769 PMC3967030

[B31] Jackson S. Nicolson S. W. (2002). Xylose as a nectar sugar: from biochemistry to ecology. Comp. Biochem. Physiol. 131, 613–620. 10.1016/S1096-4959(02)00028-3 11923077

[B32] Kaczorowski R. L. Gardener M. C. Holtsford T. P. (2005). Nectar traits in *Nicotiana* section *Alatae* (Solanaceae) in relation to floral traits, pollinators and mating system. Am. J. Bot. 92, 1270–1283. 10.3732/ajb.92.8.1270 21646148

[B33] Kram B. W. Xu W. W. Carter C. J. (2009). Uncovering the *Arabidopsis thaliana* nectary transcriptome: investigation of differential gene expression in floral nectariferous tissues. BMC Plant Biol. 9:92. 10.1186/1471-2229-9-92 19604393 PMC2720969

[B34] Lehmann S. Gumy C. Blatter E. Boeffel S. Fricke W. Rentsch D. (2011). In planta function of compatible solute transporters of the AtProT family. J. Exp. Bot. 62, 787–796. 10.1093/jxb/erq320 20959625 PMC3003823

[B35] Mariani C. De Beuckeleer M. Truettner J. Leemans J. Goldberg R. B. (1990). Induction of male sterility in plants by a chimeric ribonuclease gene. Nature 357, 737–741. 10.1038/347737a0

[B36] Mattioli R. Biancucci M. Lonoce C. Costantino P. Trovato M. (2012). Proline is required for male gametophyte development in Arabidopsis. BMC Plant Biol. 12:236. 10.1186/1471-2229-12-236 23234543 PMC3543202

[B37] Mattioli R. Falasca G. Sabatini S. Altamura M. M. Costantino P. Trovato M. (2009). The proline biosynthetic genes *P5CS1* and *P5CS2* play overlapping roles in Arabidopsis flower transition but not in embryo development. Physiol. Plant. 137, 72–85. 10.1111/j.1399-3054.2009.01261.x 19627555

[B38] Mattioli R. Marchese D. D'Angeli S. Altamura M. M. Costantino P. Trovato M. (2008). Modulation of intracellular proline levels affects flowering time and inflorescence architecture in *Arabidopsis*. Plant Mol. Biol. 66, 277–288. 10.1007/s11103-007-9269-1 18060533

[B39] Mayfield J. A. Fiebig A. Johnstone S. E. Preuss D. (2001). Gene families from the *Arabidopsis thaliana* pollen coat proteome. Science 292, 2482–2485. 10.1126/science.1060972 11431566

[B40] Mazzucotelli E. Tartari A. Cattivelli L. Forlani G. (2006). Metabolism of γ-aminobutyric acid during cold acclimation and freezing and its relationship to frost tolerance in barley and wheat. J. Exp. Bot. 57, 3755–3766. 10.1093/jxb/erl141 16997899

[B41] Mevi-Schutz J. Erhardt A. (2005). Amino acids in nectar enhance butterfly fecundity: a long-awaited link. Am. Nat. 165, 411–420. 10.1086/429150 15791533

[B42] Michaeli S. Fait A. Lagor K. Nunes-Nesi A. Grillich N. Yellin A.. (2011). A mitochondrial GABA permease connects the GABA shunt and the TCA cycle, and is essential for normal carbon metabolism. Plant J. 67, 485–498. 10.1111/j.1365-313X.2011.04612.x 21501262

[B43] Michaeli S. Fromm H. (2015). Closing the loop on the GABA shunt in plants: are GABA metabolism and signaling entwined? Front. Plant Sci. 6:419. 10.3389/fpls.2015.00419 26106401 PMC4460296

[B44] Micheu S. Crailsheim K. Leonhard B. (2000). Importance of proline and other amino acids during honeybee flight (*Apis mellifera carnica* POLLMANN). Amino Acids 18, 157–175. 10.1007/s007260050014 10817408

[B45] Mollet J. C. Park S. Y. Nothnagel E. A. Lord E. M. (2000). A lily stylar pectin is necessary for pollen tube adhesion to an *in vitro* stylar matrix. Plant Cell 12, 1737–1750. 10.1105/tpc.12.9.1737 11006344 PMC149082

[B46] Mutters R. G. Ferreira L. G. R. Hall A. E. (1989). Proline content of the anthers and pollen of heat-tolerant and heat-sensitive cowpea subjected to different temperatures. Crop Sci. 29, 1497–1500. 10.2135/cropsci1989.0011183X002900060036x

[B47] Nakashima K. Satoh R. Kiyosue T. Yamaguchi-Shinozaki K. Shinozaki K. (1998). A gene encoding proline dehydrogenase is not only induced by proline and hypoosmolarity, but is also developmentally regulated in the reproductive organs of *Arabidopsis*. Plant Physiol. 118, 1233–1241. 10.1104/pp.118.4.1233 9847097 PMC34739

[B48] Naqvi S. M. S. Harper A. Carter C. Ren G. Guirgis A. York W. S.. (2005). Nectarin IV, a potent endoglucanase inhibitor secreted into the nectar of ornamental tobacco plants. Isolation cloning and characterization. Plant Physiol. 139, 1389–1400. 10.1104/pp.105.065227 16244157 PMC1283774

[B49] Okuda S. Suzuki T. Kanaoka M. Mori H. Sasaki N. Higashiyama T. (2013). Acquisition of LURE-binding activity at the pollen tube tip of *Torenia fournieri*. Mol. Plant 6, 1074–1090. 10.1093/mp/sst050 23482369

[B50] Okuda S. Tsutsui H. Shiina K. Sprunck S. Takeuchi H. Yui R.. (2009). Defensin-like polypeptide LUREs are pollen tube attractants secreted from synergid cells. Nature 458, 357–361. 10.1038/nature07882 19295610

[B51] Olsen R. W. Sieghart W. (2008). International Union of Pharmacology LXX. Subtypes of γ-aminobutyric acidA receptors: classification on the basis of subunit composition, pharmacology, and function. Update. Pharmacol. Rev. 60, 243–260. 10.1124/pr.108.00505 18790874 PMC2847512

[B52] Palanivelu R. Brass L. Edlund A. F. Preuss D. (2003). Pollen tube growth and guidance is regulated by *POP2*, an *Arabidopsis* gene that controls GABA levels. Cell 114, 47–59. 10.1016/S0092-8674(03)00479-3 12859897

[B53] Peumans W. J. Smeets K. Van Nerum K. Van Leuven F. Van Damme E. J. M. (1997). Lectin and alliinase are the predominant proteins in nectar from leek (*Allium porrum* L.) flowers. Planta 201, 298–301. 10.1007/s004250050070 9129337

[B54] Pyke G. H. (1991). What does it cost a plant to produce floral nectar? Nature 350, 58–59. 10.1038/350058a0

[B55] Qin Y. Leydon A. R. Manziello A. Pandey R. Mount D. Denic S.. (2009). Penetration of the stigma and style elicits a novel transcriptome in pollen tubes, pointing to genes critical for growth in a pistil. PLOS Genet. 5:e1000621. 10.1371/journal.pgen.1000621 19714218 PMC2726614

[B56] Radhika V. Kost C. Boland W. Heil M. (2010). The role of jasmonates in floral nectar secretion. PLoS ONE 5:e9265. 10.1371/journal.pone.0009265 20174464 PMC2824824

[B57] Ramesh S. A. Tyerman S. D. Xu B. Bose J. Kaur S. Conn V.. (2015). GABA signalling modulates plant growth by directly regulating the activity of plant-specific anion transporters. Nat Commun. 6, 7879. 10.1038/ncomms8879 26219411 PMC4532832

[B58] Renault H. El Amrani A. Berger A. Mouille G. Soubigou-Taconnat L. Bouchereau A.. (2013). γ-aminobutyric acid transaminase deficiency impairs central carbon metabolism and leads to cell wall defects during salt stress in *Arabidopsis* roots. Plant Cell Environ. 36, 1009–1018. 10.1111/pce.12033 23148892

[B59] Rentsch D. Hirner B. Schmelzer H. Frommer W. (1996). Salt stress-induced proline transport and salt stress-repressed broad specificity amino acid permeases ldentified by suppression of a yeast amino acid permease-targeting mutant. Plant Cell 8, 1437–1446. 10.1105/tpc.8.8.1437 8776904 PMC161269

[B60] Ruhlmann J. M. Kram B. W. Carter C. J. (2010). *CELL WALL INVERTASE 4* is required for nectar production in Arabidopsis. J. Exp. Bot. 61, 395–404. 10.1093/jxb/erp309 19861655 PMC2803206

[B61] Sangwan R. S. (1978). Change in the amino-acid content during male gametophyte formation of *Datura metel in situ*. Theor. Appl. Genet. 52, 221–225. 10.1007/BF00273893 24317576

[B62] Savouré A. Jaoua S. Hua X. J. Ardiles W. Van Montagu M. Verbruggen N. (1995). Isolation and characterization, and chromosomal location of a gene encoding the δ^1^-pyrroline-5-carboxylate synthetase in *Arabidopsis*. FEBS Lett 372, 13–19. 10.1016/0014-5793(95)00935-3 7556633

[B63] Schwacke R. Grallath S. Breitkreuz K. E. Stransky H. Frommer W. B. Rentsch D. (1999). LeProT1, a transporter for proline, glycine betaine, and γ-amino butyric acid in tomato pollen. Plant Cell 11, 377–391. 10.2307/3870867 10072398 PMC144187

[B64] Shelp B. J. Bown A. W. McLean M. D. (1999). Metabolism and functions of gamma-aminobutyric acid. Trends Plant Sci. 4, 446–452. 10.1016/S1360-1385(99)01486-7 10529826

[B65] Strizhov N. Ábrahám E. Ökrész L. Blickling S. Zilberstein A. Schell J.. (1997). Differential expression of two P*5CS* genes controlling proline accumulation during salt-stress requires ABA and is regulated by ABA1, ABI1 and AXR2 in Arabidopsis. Plant J. 12, 557–569. 10.1111/j.0960-7412.1997.00557.x 9351242

[B66] Suárez C. Zienkiewicz A. Castro A. J. Zienkiewicz K. Majewska-Sawka A. Rodríguez-García M. I. (2013). Cellular localization and levels of pectins and arabinogalactan proteins in olive (*Olea europaea* L.) pistil tissues during development: implications for pollen-pistil interaction. Planta 237, 305–319. 10.1007/s00425-012-1774-z 23065053

[B67] Szabados L. Savouré A. (2010). Proline: a multifunctional amino acid. Trends Plant Sci. 15, 89–97. 10.1016/j.tplants.2009.11.009 20036181

[B68] Székely G. Ábrahám E. Cséplo Á. Rigo G. Zsigmond L. Csiszár J.. (2008). Duplicated *P5CS* genes of *Arabidopsis* play distinct roles in stress regulation and developmental control of proline biosynthesis. Plant J. 53, 11–28. 10.1111/j.1365-313X.2007.03318.x 17971042

[B69] Terrab A. Garcia-Castano J. L. Romero J. M. Berjano R. De Vega C. Talavera S. (2007). Analysis of amino acids in nectar from *Silene colorata* Poiret (Caryophyllaceae). Bot. J. Linn. Soc. 155, 49–56. 10.1111/j.1095-8339.2007.00673.x

[B70] Thornburg R. W. Carter C. Powell A. Mittler R. Rizhsky L. Horner H. T. (2003). A major function of the tobacco floral nectary is defense against microbial attack. Plant Syst. Evol. 238, 211–218. 10.1007/s00606-003-0282-9

[B71] Trovato M. Mattioli R. Costantino P. (2008). Multiple roles of proline in plant stress tolerance and development. Rend. Lincei 19, 325–346. 10.1007/s12210-008-0022-826106823

[B72] Vansuyt G. Vallee J. C. Prevost J. (1979). La pyrroline-5-carboxylate réductase et la proline déhydrogénase chez *Nicotiana tabacum* var. Xanthi n.c. en fonction de son développement. Physiol. Veg. 19, 95–105.

[B73] Venekamp J. H. Koot J. T. M. (1988). The sources of free proline and asparagine in field bean plants, *Vicia faba* L., during and after a short period of water withholding. J. Plant Physiol. 32, 102–109. 10.1016/S0176-1617(88)80192-5

[B74] Verbruggen N. Hermans C. (2008). Proline accumulation in plants: a review. Amino Acids 35, 753–759. 10.1007/s00726-008-0061-6 18379856

[B75] Vogler F. Schmalzl C. Englhart M. Bircheneder M. Sprunck S. (2014). Brassinosteroids promote *Arabidopsis* pollen germination and growth. Plant Reprod. 27, 153–167. 10.1007/s00497-014-0247-x 25077683

[B76] Walton E. F. Clark C. J. Boldingh H. L. (1991). Effect of hydrogen cyanamide on amino acid profiles in kiwifruit buds during bud-break. Plant Physiol. 97, 1256–1259. 10.1104/pp.97.3.1256 16668518 PMC1081151

[B77] Wilhelmi L. K. Preuss D. (1996). Self-sterility in *Arabidopsis* due to defective pollen tube guidance. Science 274, 1535–1537. 10.1126/science.274.5292.1535 8929415

[B78] Wolters-Arts M. Lush W. M. Mariani C. (1998). Lipids are required for directional pollen-tube growth. Nature 392, 818–821. 10.1038/33929 9572141

[B79] Wu H. M. Wong E. Ogdahl J. Cheung A. Y. (2000). A pollen tube growth- promoting arabinogalactan protein from *Nicotiana alata* is similar to the tobacco TTS protein. Plant J. 22, 165–176. 10.1046/j.1365-313x.2000.00731.x 10792832

[B80] Yoshiba Y. Kiyosue T. Katagiri T. Ueda H. Wada K. Harada Y.. (1995). Correlation between the induction of a gene for δ^1^-pyrroline-5-carboxylate synthetase and the accumulation of proline in *Arabidopsis* under osmotic stress. Plant J. 7, 751–760. 10.1046/j.1365-313X.1995.07050751.x 7773306

[B81] Yu G. H. Zou J. Feng J. Peng X. B. Wu J. Y. Wu Y. L.. (2014). Exogenous γ-aminobutyric acid affects pollen tube growth via modulating putative Ca^2+^-permeable membrane channels and is coupled to negative regulation on glutamate decarboxylase. J. Exp. Bot. 12, 3235–3248. 10.1093/jxb/eru171 24799560 PMC4071839

